# Personality and well-being in adolescents

**DOI:** 10.3389/fpsyg.2014.01494

**Published:** 2015-01-07

**Authors:** Paulo A. S. Moreira, C. Robert Cloninger, Liliana Dinis, Laura Sá, João T. Oliveira, Adelaide Dias, Joana Oliveira

**Affiliations:** ^1^Instituto de Psicologia e de Ciências da Educação, Universidade Lusíada do PortoPorto, Portugal; ^2^Center for Well-being, School of Medicine, Washington UniversitySt. Louis, MO, USA

**Keywords:** personality, character, adolescents, psychobiological model of personality, wellbeing, health, wellness, happiness

## Abstract

Different profiles of the character dimensions of self-directedness, cooperativeness and self-transcendence result in different levels of wellbeing among adults. However, the influence of the multidimensional character profiles on adolescents' composite wellbeing remains unexplored. This study builds on previous studies with adults, and examines the linear and non-linear associations between the dimensions of the psychobiological model of personality and well-being in adolescents. Participated in this study 1540 adolescents (*M* = 15.44, *SD* = 1.731). Personality was assessed using the Temperament and Character Inventory (TCI). Well-being was evaluated in a composite perspective: satisfaction with social support, health-related quality of life, satisfaction with life and affect. Variable-centered and individual-centered analyses were performed. Self-directedness was strongly associated with all dimensions of affective and cognitive well-being regardless of the other two character traits. Cooperativeness was associated with non-affective well-being and with positive affect, but only when associated to elevation of Self-directedness and Self-transcendence. Self-Directedness and Cooperativeness explained 15.5% of the non-affective well-being variance. Self-Directedness and Self-Transcendence explained 10.4% of the variance in affective well-being. This study confirms the tendencies found in previous studies with adults from other societies, where each character dimension gives an independent contribution to well-being depending on the interactions with other Character dimensions. Also, this study highlights the importance of considering the non-linear influences of the character dimensions in understanding of adolescents' wellbeing. These results have strong implications for youth positive mental health promotion, including for school-based policies and practices.

This study builds on research developed by Cloninger and Zohar (2011) and Josefsson et al. (2011) with adult populations, by describing the non-linear influences of character profiles on wellbeing in adolescents.

Adolescents well-being is highly associated to several indicators of developmental trajectories (Pyhältö et al., 2010), including engagement with school (Elmore and Huebner, 2010; Ainly and Ainly, 2011; Lewis et al., 2011), academic achievement (Berger et al., 2011), optimism and coping strategies, and is a protective factor against negative indicators of health (Carver et al., 2010). Adolescents with high levels of well-being are more resilient (Gilman and Huebner, 2006; Antaramian et al., 2010), present lower delinquency behaviors and aggression, lower depressive and anxiety symptoms, higher self-esteem, self-efficacy and adaptation (McKnight et al., 2002; Huebner, 2004; Suldo and Huebner, 2004; Antaramian et al., 2010).

Well-being is a multidimensional phenomenon, integrating biological, psychological, social, and spiritual dimensions (Cloninger, 2004, 2006a,b; Lyubomirsky et al., 2005; Bartels and Boomsma, 2009; McDowell, 2010). Wellbeing refers to the emotional and cognitive dimensions of the subjective experience resulting from the individual evaluation of several dimensions of life. Conceptions of well-being vary from Hedonic and Eudaimonic distinct but related and complementary approaches (Keyes et al., 2002; Huppert and Whittington, 2003). Hedonic well-being refers to the emotional dimensions of the individuals' positive life experiencing (Diener, 1984), including absence of negative emotions, presence of positive emotions, life satisfaction and social involvement (Ryan and Deci, 2001). Eudaimonic well-being refers to the harmony between the individuals goals and values and life experiences (Ryff et al., 2004), and is associated to individuals personal development (Ryan and Deci, 2001).

Personality is a significant predictor of mental health (Cloninger et al., 1997; Gestsdóttir and Lerner, 2007; Davydov et al., 2010), including positive mental health/wellbeing (Cloninger and Zohar, 2011; Josefsson et al., 2011; Butkovic et al., 2012). Healthy personality development is related to several aspects of well-being and there is a need for integrating the contributions of personality to well-being on current approaches to mental health (Seligman, 2008; Cloninger, 2012; Vaillant, 2012). Studies using personality models derived from linear factor analyses, such as the Five-Factor Model (FFM) (McCrae and Costa, 1991; Gutiérrez et al., 2005), found negative associations between Neuroticism and happiness and psychological wellbeing (Stewart et al., 2005; Garcia, 2011), positive associations between Neuroticism and negative affect, between Openness and positive affect and between Conscientiousness and life satisfaction (DeNeve and Cooper, 1998; Garcia, 2011). Extraversion was found to be positively related to positive affect (Diener et al., 2003; Lyubomirsky et al., 2006; Garcia, 2011). Eysenck's dimension of Extraversion was found to be associated to happiness and to loneliness and Neuroticism was negatively correlated to happiness (Cheng and Furnham, 2002). Mixed results of positive relation (Huebner et al., 2004) and absence of relation (Rigby and Huebner, 2005) have been found for the relation between Extraversion and life satisfaction among and adolescents.

However, there is a growing consensus about the need of using genetic-informed and psychobiological personality models, as they are more adequate for describing psychobiological processes underlying behavior than lexical models (Cloninger, 2008b; de Moor et al., 2010; Munafò and Flint, 2011; Veselka et al., 2012).

Cloninger and colleagues developed the psychobiological model of personality which conceptualizes personality as an organization of dynamic and non-linear psychobiological processes (Cloninger et al., 1993). The authors developed age-appropriated instruments of the Temperament and Character Inventory (TCI), which measures temperament and character dimensions. Temperament refers to individual differences in behavioral conditioning of responses to basic emotional stimuli related to fear, anger, disgust, and ambition. There are 4 TCI temperament dimensions: Novelty Seeking (NS) (i.e., impulsive vs. deliberate); Harm Avoidance (HA) (i.e., anxious vs. risktaking); Reward Dependence (RD) (i.e., sociable vs. aloof), and Persistence (PS) (i.e., determined vs. easily discouraged). Each extreme of temperament has advantages and disadvantages depending on the situation (Cloninger et al., 1993; Cervone, 2005). Character refers to individual differences in higher order socio-cognitive processes (self-concepts, and intentional values and goals) (Cloninger, 2008a). The 3 dimensions of TCI character are called Self-Directedness (SD) (i.e., purposeful vs. aimless), Cooperativeness (CO) (i.e., helpful vs. hostile), and Self-Transcendence (ST) (i.e., holistic vs. self-centered) (Cloninger et al., 1993). Because Temperament refers to the tendency of responding to basic emotional stimuli, it is more strongly related to hedonic well-being (Cloninger et al., 1998). High levels of Extroversion of the Five-Factor Model (which corresponds to low scores of the psychobiological model personality dimension of HA, Cloninger, 2010) tend to be more respondents to positive affect (Larsen and Eid, 2008). Also, high levels of Neuroticism (which corresponds to low persistence and low self-directedness (Cloninger, 2010) are associated to more reactivity to negative affect (Larsen and Eid, 2008). These results are consistent to those found in adolescents, where high levels of Harm Avoidance predicted low levels of Positive Affect (Garcia, 2011). By another hand, Character refers to higher order socio-cognitive self-regulatory processes, and is more associated to the Eudaimonic well-being (Cloninger, 2004). Both Temperament and Character are associated to physical and emotional health, although the evidences for the associations between temperament and health are less consistent (Ryff et al., 2004; Westerhof and Keyes, 2010).

Two recent population-based studies in Israel and Finland used the multidimensional psychobiological personality profiles to assess the linear and non-linear effects of interactions among dimensions on different indicators of well-being (Cloninger and Zohar, 2011; Josefsson et al., 2011). Character dimensions of self-directedness, cooperativeness and self-transcendence shown to be strong predictors of the different aspects of well-being. In the Israeli population-based study Self-directedness was strongly correlated with affective (positive and negative affect) and non-affective (life satisfaction, social support and subjective health) dimensions of well-being. Cooperativeness was especially associated to satisfaction with social support and Self-transcendence predicted positive emotions (Cloninger and Zohar, 2011). Similar findings were found in the Finn population-based study, where personality explained half the variance in non-affective aspects of well-being and two thirds of the variance in affective dimensions of well-being (Josefsson et al., 2011). Besides, each character dimension independently contributes to well-being, depending of interactions among dimensions, which means that the character profiles are strongly associated with individual differences in well-being (Josefsson et al., 2011). However, in the Finn study, Self-Transcendence was associated with both positive and negative affect, while in the Israeli study it was only associated with positive affect, which suggest that the effect of Self-transcendence on well-being depends on cultural and religion differences (Josefsson et al., 2011).

Adolescence is a developmental period characterized by marked transformations in psychobiological processes underlying behaviors, due to the maturation of the neuroanatomical circuitries, the specificities of the contexts and the development tasks associated (autonomy, intimacy, etc.). Adolescents' psychobiological organizations are modulated by interactions among individual and context dimensions resulting in different pattern of functioning, from positive to negative functioning. Although personality development is characterized by continuity, temperament and character dimensions have different development patterns (Josefsson et al., 2013a), with character dimensions exerting a significant influence on individuals functioning, including on wellbeing (Cloninger and Zohar, 2011; Josefsson et al., 2011). In spite of its importance to the promotion of adolescents positive functioning, the influences of different combinations of character dimensions on adolescents' wellbeing remain unexplored. Childhood personality is a significant predictor of competence and resilience in adulthood (Shiner and Masten, 2012), and dimensions of positive mental health systematized by Vaillant (2012) are involved in cascades of children and adolescents positive and negative development (Blandon et al., 2010; Bornstein et al., 2010). Because well-being is a central dimension on positive development cascades (Lewin-Bizan et al., 2010), a developmental approach to mental health requires the understanding of the developmental associations between the psychobiological processes underlying personality and well-being also in earlier stages of development, including early and middle adolescence. In addition, a fully understanding of adolescents health requires the use of genetic-, neuroanatomic, and psychological-informed frameworks (Burnett et al., 2011; Sturman and Moghaddam, 2011; Eldreth et al., 2013; Richards et al., 2013).

Several authors are arguing that Character dimensions need to be considered in the understanding of the associations between personality and well-being (Cloninger et al., 2010; Cloninger and Zohar, 2011; Garcia, 2011; Garcia and Moradi, 2011). Recent studies conducted by Garcia and colleagues found that dimensions of the psychobiological model of personality are strong predictors of adolescents wellbeing. Different temperament and character dimensions registered different associations with wellbeing. Self-directedness showed to be the most important predictor of adolescents' wellbeing, also because it mediated the relationship between temperament dimensions (e.g., Persistence) and wellbeing (Garcia, 2011; Garcia and Moradi, 2011; Garcia et al., 2012).

Rather than a linear phenomenon, the development of well-being encompasses complexes and non-linear interactions between personality dimensions involved in adaptation. Healthy personality development depends on the growth in self-awareness (Cloninger, 2008a) and on the differentiation of dimensions such as strengths of character, maturity, positive emotional balance, socio-emotional intelligence, life satisfaction (true happiness), and resilience (Vaillant, 2012). The same personality dimensions can result in different outcomes (i.e., multi-finality), and different configurations of personality dimensions can lead to the same outcome (i.e., equifinality) (Cloninger and Cloninger, 2011). Therefore, because of the complexity of developmental psychobiological processes, a fully understanding of effects of personality dimensions on well-being requires person-centered approaches, because it allows for the understanding of how different personality profiles (rather than separate dimensions) affect the individuals' subjective experience.

As described by previous studies with adults conducted by Cloninger and colleagues, the personality influences on wellbeing are better described throughout non-linear associations and combinations between different temperament and character dimensions, rather than linear associations only (Cloninger and Zohar, 2011; Josefsson et al., 2011). In spite of that, no study had evaluated the non-linear associations of Character dimensions of self-directedness, cooperativeness and self-transcendence with well-being in adolescents, which are significant predictors of physical, mental, and social components of health and happiness (Cloninger and Zohar, 2011).

The objective of this study was to build on Cloninger and Zohar (2011) and on Josefsson et al. (2011) by describing the non-linear associations between Cloninger' psychobiological model of personality multidimensional character profiles (self-directedness, cooperativeness and self-transcendence) and well-being (measured as a composite indicator of satisfaction with social support, life satisfaction, health-related quality of life and affect) in adolescents.

## Methods

### Participants

Participated in this study 1540 Portuguese adolescents aged 12–21 years old (*M* = 15.44, *SD* = 1.731). Adolescents were nearly equally divided by gender (45.2% male; 51.7% female). Those students who did not include the information about the gender were not included in this descriptive of participants' gender. Participants were also nearly divided by school level (53.8% Middle School; 46.2% Secondary School). The majority of adolescents were enrolled in regular schools (*n* = 1197, 77.7%) and the others (*n* = 343, 22.3%) in vocational schools.

### Measures

Socio-demographics—Socio-demographic characteristics of adolescents, such as age, parent and mother education, parent and mother occupation, were collected. Students filled out the required socio-economic in the socio-demographics inventory. However, a very substantial proportion of the students did not give information about parents' occupational status, and it was not possible to collect the family's annual/monthly incomes. Because parental education (and especially maternal education) is the strongest predictor of family SES, and it is an acceptable indicator of SES (Bradley and Corwyn, 2002), we considered parents education as the indicator for SES status. Mothers educational attainment in our sample was as follows: 20.7% completed only the 4th grade; 23.2% completed only the 6th grade; 18.6% completed only the 9th grade; 11.8% completed only the 12th grade and 25.7% had a graduation or post-graduation). The fathers' education was similar: 21.8% completed only the 4th grade; 21.7% completed the 6th grade; 17.8% completed only the 9th grade; 12% completed the 12th grade and 26.7% had a graduation or post-graduation. In both mothers and fathers, the percentage of those who had only completed the 6th grade (44% of mothers and 43.5% of fathers) was almost twice those who had a graduation (25.7% of mothers and 26.7% of the fathers).

#### TCI-R

The Temperament and Character Inventory—Revised (TCI-R; Cloninger, 1999) is a comprehensive personality inventory for adults aged 17 and older. It has 240-items rated by a 5-point Likert response format (Completely False to Completely True). It measures 4 dimensions of temperament—Novelty Seeking (NS), Harm Avoidance (HA), Reward Dependence (RD) and Persistence (PS)—and 3 of character—Self-Directedness (SD), Cooperativeness (CO) and Self-Transcendence (ST)—comprised of 29 subscales. The TCI-R Portuguese version has a good internal consistency for all the dimensions with coefficient values for Cronbach *alpha* above 0.84, except for Novelty Seeking (NS) and Reward Dependence (RD) (0.79 and 0.80, respectively) (Moreira et al., in preparation).

#### JTCI

The Junior Temperament and Character Inventory (JTCI; Luby et al., 1999) is a 108 item inventory for parent-report, teacher-report, or self-report, and it uses a true-false format to simplify responses in younger children. The Junior Temperament and Character Inventory (JTCI) measures the 7 major dimensions of the psychobiological model of Temperament and Character, throughout age-appropriate items corresponding to all the adult TCI scales. In the validity based studies of the Portuguese version of the JTCI, 2 modifications were made to the American version: (1) all items were rated on a 5-point Likert scale with 5 options (1 = completely False, 2 = mostly False, 3 = cannot decide, 4 = mostly True, and 5 = completely True); (2) 16 additional items were added or changed, in order to better accommodate cultural specificities [2 items were added to the Reward Dependence and 9 items were added to the Self-Transcendence scale (5) items to the subscale of Fantasy and Imagination (ST1) and (4) items to the Spirituality subscale (ST2)]. These changes were made in accordance with the author of the instrument, and they did not change the constructs of the dimensions. The JTCI Portuguese version has 127 items, and has moderate to strong internal consistency for all dimensions: Novelty Seeking: α = 0.77; Harm Avoidance: α = 0.83; Reward Dependence: α = 0.62; Persistence: α = 0.50; Self-Directedness; α = 0.75; Cooperativeness; α = 0.78; and Self-Transcendence: α = 0.69 (Moreira et al., 2012b).

#### Character profiles

In order to describe the non-linear influences of different character dimensions combinations, we relied on Cloninger's proposal for 8 character profiles: SCT (Creative profile; elevation on the 3 dimensions of Self-Directedness); SCt (Organized profile; elevation on Self-Directedness and Cooperativeness and low scores on Self-Transcendence); ScT (Fanatical profile; High Self-Directedness and Self-Transcendence and low Cooperativeness); Sct (Autocratic profile; High Self-Directedness and low Cooperativeness and Self-Transcendence); sCT (Moody profile; low Self-Directedness and high Cooperativeness and Self-Transcendence); sCt (Dependent profile; low Self-Directedness and Self-Transcendence and high Cooperativeness); scT (Disorganized profile; low Self-Directedness and Cooperativeness and high Self-Transcendence); and sct (Depressive profile; low scores in the 3 dimensions of Self-Directedness, Cooperativeness and Self-Transcendence) (Cloninger, 2004; Cloninger and Zohar, 2011; Josefsson et al., 2011). As in previous studies, the participants were distributed in 2 groups: those presenting scores above the mean, and those presenting scores below the mean for each of the character dimensions. Then, they were grouped in the 8 possible combinations of profiles (Table 1) (Cloninger, 2004; Cloninger and Zohar, 2011; Josefsson et al., 2011).

**Table 1:** **Frequency distribution of the TCI (measured both with the JTCI and the TCI-R versions) character profiles**.

**Character profile**	**N**	**Valid (%)**
sct—depressive	327	21.20
scT—disorganized	212	13.80
sCt—dependent	111	7.20
sCT—moody	155	10.10
Sct—autocratic	150	9.70
ScT—fanatical	79	5.10
SCt—organized	261	17
SCT—creative	244	15.90

#### Life satisfaction

To assess the life satisfaction we used the Brief Multidimensional Students' Life Satisfaction Scale (Huebner et al., 2011). It includes six items that assesses six different domains of life (Family, Friends, School, Self, Environment, Life in general) by a seven-point Likert-like scale (Terrible; Unhappy; Unsatisfactory; Partly unsatisfactory and Partly satisfactory; Satisfactory; Friendly, Fantastic).

#### Social support

The social support was assessed by the Portuguese version of the Brief Version of the Satisfaction with Social Support Scale for Children and Adolescents (Gaspar et al., 2009a,b). The scale includes six items that assesses the satisfaction with social support (e.g., “I am satisfied with the amount of friends I have”) and six items that assesses the need for activities related to social support (e.g., “My friends do not come to me as often as I liked”). The items are rated on a five-point scale. Scale reliability was α = 0.70.

#### Affect

The Positive and Negative Affect Scale (PANAS, Watson et al., 1998) was used to assess the positive and negative states which are endorsed on a five-point Likert-like scale. In our study, the scale registered good internal consistency values (with alphas of 0.90 and 0.92 for positive and negative affect scales, respectively), similar to those found in the study of the Psychometric characteristics of the Portuguese version (Galinha and Pais-Ribeiro, 2005).

#### Quality of life

The health-related quality of life was assessed with KIDSCREEN-10 (Erhart et al., 2009). The KIDSCREEN-10 is a brief instrument that assesses mental health and well-being in children/adolescents aged between 8 and 18 years. It includes 10 items (e.g., “Felt fit and well”; “Felt full of energy”; “felt sad”) answered on a Likert scale with five response options (from 1—“never” to 5—“always”). The Portuguese version (Matos et al., 2012) has good psychometric characteristics with a internal consistency of 0.78.

#### Composite health index and happiness index

In order to examine the associations between character profiles and the two higher order dimensions of wellbeing, we estimated the index of Composite Health and of Happiness as indicators of non-affective (wellness) and affective (happiness) wellbeing, respectively. We relied on the proposals of Cloninger and Zohar (2011), and of Josefsson et al. (2011) for this estimation. The Composite Health Index refers to the mean of the Satisfaction with social support, Satisfaction with life, and Health related quality of life. The Happiness Index was estimated as the score of the Positive affect minus the score of the Negative affect; it reflects, therefore, the emotional tonality of the individuals' experience: the salience of the positive emotions (desirably present) and of the negative emotions (desirably absence).

### Procedure

#### Data collection

The individuals were recruited accordingly to the snow ball technique for the selection of non-randomized samples. Adolescents were contacted by researchers in the school context. All students were asked to deliver the informed consent to their parents, so they could decide if they will consent their adolescent to take part of the study. Besides parents' informed consent, 18 older or less were also asked if they wanted to participate in the study. Adolescents with 18 years or more were asked to sign in the informed consent. Those adolescents who brought the informed consent signed in by their parents (under 18) or by themselves (adolescents with 18 years old or more), and who wanted to participate in the study were gathered in a group session of 1 h, in classrooms. Then the socio-demographic, the wellbeing and the personality questionnaires were distributed to students. For 17 olders or less, the JTCI was distributed, for 18 older or more the TCI-R was distributed. No extra time was needed, and some discomfort was observed from some participants. Adolescents were reminded that they could drop out without completing the questionnaires. This only happened in a few cases (who were not included in the study), and the great majority of the participants did not express any disturbance or discomfort. In order to protect the participants' identity, the questionnaires were precoded by the researchers with a code for each school, for school year, and for student. Then the researchers distributed the questionnaires already precoded.

#### Statistical analysis

All data were carefully double-checked for possible miscoding, distribution of values, and updating of missing values prior to analysis (some items had missing data, and we replaced them by the series mean method). In order to assess the non-linear associations between personality configurations and well-being, character profiles were defined. The participants were grouped according to all the possible combinations of high and low scores in each one of the character dimensions. Our non-linear analyses were based on the Cloninger's proposal for character profiles (Cloninger, 2004; Cloninger and Zohar, 2011; Josefsson et al., 2011). Pearson's correlations, principal components analysis, multiple regression analyses and *t*-tests were all carried out using the Statistical Package for Social Sciences (SPSS) for Windows, version 18.0.

## Results

### Personality dimensions and well-being by school level and curriculum type

Personality dimensions, measures of well-being, Happiness Index (HI) and Composite Health Index (CHI) were examined by curriculum type (Table 2) and school level (Table 3). Middle school students presented higher Novelty Seeking (*t* = 3.56; *p* = 0.00), Self-Transcendence (*t* = 4.93; *p* = 0.00), Life Satisfaction (*t* = 2.12; *p* = 0.03), Health-related quality of life (*t* = 6.19; *p* = 0.00), Positive Affect (*t* = 5.30; *p* = 0.00), and Negative Affect (*t* = 2.30; *p* = 0.02). Conversely, Middle school students registered lower Reward Dependence (*t* = −3.26; *p* = 0.00), Persistence (*t* = −3.15; *p* = 0.00), Self-Directedness (*t* = −6.52; *p* = 0.00), Cooperativeness (*t* = −5.22; *p* = 0.00) and Social Support (*t* = −2.67; *p* = 0.01) comparatively with High school students.

**Table 2:** **TCI dimensions (measured both with the JTCI and the TCI-R versions), measures of well-being, Happiness Index and Health Index by school level**.

	**Middle School (*n* = 829)**		**High School (*n* = 711)**		***t***	***p***	***Cohen's d***	***Effect size r***
	**Mean**	***SD***	**Mean**	***SD***				
Novelty Seeking	0.08	1.03	−0.09	0.96	3.56	0.00	0.18	0.09
Harm Avoidance	−0.02	0.97	0.03	1.03	−0.89	0.38	−0.04	−0.02
Reward Dependence	−0.08	0.97	0.09	1.03	−3.26	0.00	−0.17	−0.08
Persistence	−0.07	0.97	0.09	1.03	−3.15	0.00	−0.16	−0.08
Self-Directedness	−0.15	1.00	0.18	0.97	−6.52	0.00	−0.33	−0.16
Cooperativeness	−0.12	1.01	0.14	0.96	−5.22	0.00	−0.27	−0.13
Self-Transcendence	0.12	0.99	−0.13	0.99	4.93	0.00	0.25	0.12
Life Satisfaction	0.05	1.03	−0.06	0.97	2.12	0.03	0.11	0.05
Health-related quality of life	0.14	1.05	−0.17	0.91	6.19	0.00	0.32	0.16
Positive Affect	0.12	1.04	−0.15	0.93	5.30	0.00	0.27	0.14
Negative Affect	0.05	1.09	−0.06	0.87	2.30	0.02	0.12	0.06
Social Support	−0.06	1.03	0.07	0.97	−2.67	0.01	−0.14	−0.07
Happiness Index (HI)	0.03	1.06	−0.03	0.93	1.20	0.23	0.06	0.03
Composite Health Indicator (CHI)	0.04	0.83	−0.05	0.78	2.30	0.02	0.12	0.06

*CHI (Composite Health Index) = mean of satisfaction with life, health- related quality of life and social support; HI (Happiness Index) = positive affect—negative affect*.

**Table 3:** **TCI dimensions (assessed by the JTCI and the TCI-R versions), measures of well-being, Happiness Index and Health Index by curriculum type**.

	**Regular (*n* = 1197)**		**Vocational (*n* = 343)**		***t***	***P***	***Cohen's d***	***Effect size r***
	**Mean**	***SD***	**Mean**	***SD***				
Novelty Seeking	−0.03	1.010	0.11	0.97	−2.30	0.02	−0.14	−0.07
Harm Avoidance	−0.05	1.0	0.16	0.98	−3.44	0.00	−0.21	−0.11
Reward Dependence	0.07	1.02	−0.26	0.89	5.49	0.00	0.35	0.17
Persistence	0.06	1.0	−0.21	0.97	4.54	0.00	0.28	0.14
Self-Directedness	0.07	0.98	−0.24	1.0	5.03	0.00	0.30	0.15
Cooperativeness	0.08	0.96	−0.27	1.08	5.67	0.00	0.33	0.16
Self-Transcendence	0.01	1.0	−0.02	0.99	0.57	0.57	0.04	0.02
Life Satisfaction	0.05	0.97	−0.18	1.07	3.82	0.00	0.23	0.11
Health-related quality of life	0.08	0.96	−0.28	1.09	5.99	0.00	0.36	0.17
Positive Affect	0.05	0.95	−0.19	1.13	3.91	0.00	0.23	0.11
Negative Affect	−0.04	0.97	0.15	1.10	−3.09	0.00	−0.18	−0.09
Social Support	0.06	0.99	−0.20	0.99	4.14	0.00	0.25	0.13
Happness Index (HI)	0.06	0.94	−0.20	1.14	4.30	0.00	0.25	0.12
Composite Health Indicator (CHI)	0.06	0.78	−0.22	0.88	5.76	0.00	0.35	0.17

*CHI (Composite Health Index) = mean of satisfaction with life, health-related quality of life and social support; HI (Happiness Index) = positive affect—negative affect*.

Students who attended regular schools presented higher Reward Dependence (*t* = 5.49; *p* = 0.00), Persistence (*t* = 4.54; *p* = 0.00), Self-Directedness (*t* = 5.03; *p* = 0.00), Cooperativeness (*t* = 5.63; *p* = 0.00), Life Satisfaction (*t* = 3.82; *p* = 0.00), Health-related quality of life (*t* = 5.99; *p* = 0.00), Positive Affect (*t* = 3.92; *p* = 0.00), and Social Support (*t* = 4.14; *p* = 0.00). Conversely, adolescents attending regular schools registered lower Novelty Seeking (*t* = −2.30; *p* = 0.02), Harm Avoidance (*t* = −3.44; *p* = 0.00) and Negative Affect (*t* = −3.09; *p* = 0.00) comparatively with students from vocational schools. However, the effect sizes were small for all these differences.

### Correlations between measures of well-being

The relationships among positive and negative affect, life satisfaction, perceived social support, and perceived health-related quality of life, were examined (Table 4). Positive and Negative Affectivity were weakly and negatively correlated (*r* = −0.28). The correlations between non-affective measures showed that health-related quality of life and life satisfaction were moderately correlated (*r* = 0.56), and that the social support were also moderately correlated with these measures (*r* = 0.44 and *r* = 0.49, respectively). Each individual measure of health was strongly correlated with Composite Health Index (CHI, *r* = 0.77 to *r* = 0.83). The Happiness Index was positively correlated with all indicators (*r* = 0.52 to *r* = 0.73) except with negative affect which registered a negative correlation (*r* = −0.86). The Composite Health Index (CHI) and the Happiness Index (HI) were moderately correlated (*r* = 0.66).

**Table 4:** **Correlations between measures of well-being**.

	**1**	**2**	**3**	**4**	**5**	**6**	**7**
1. Composite health Index	–						
2. Happiness Index	0.66						
3. Life Satisfaction	0.82	0.52					
4. Health-related quality of life	0.83	0.60	0.56				
5. Positive Affect	0.62	0.73	0.52	0.62			
6. Negative Affect	−0.47	−0.86	−0.34	−0.38	−0.28		
7. Social Support	0.77	0.49	0.44	0.45	0.35	−0.42	–

*CHI (Composite Health Index) = mean of satisfaction with life, health-related quality of life and social support; HI (Happiness Index) = positive affect—negative affect; All correlations are significant at p < 0.01*.

### Character profile and positive affect

**Figure 1:**
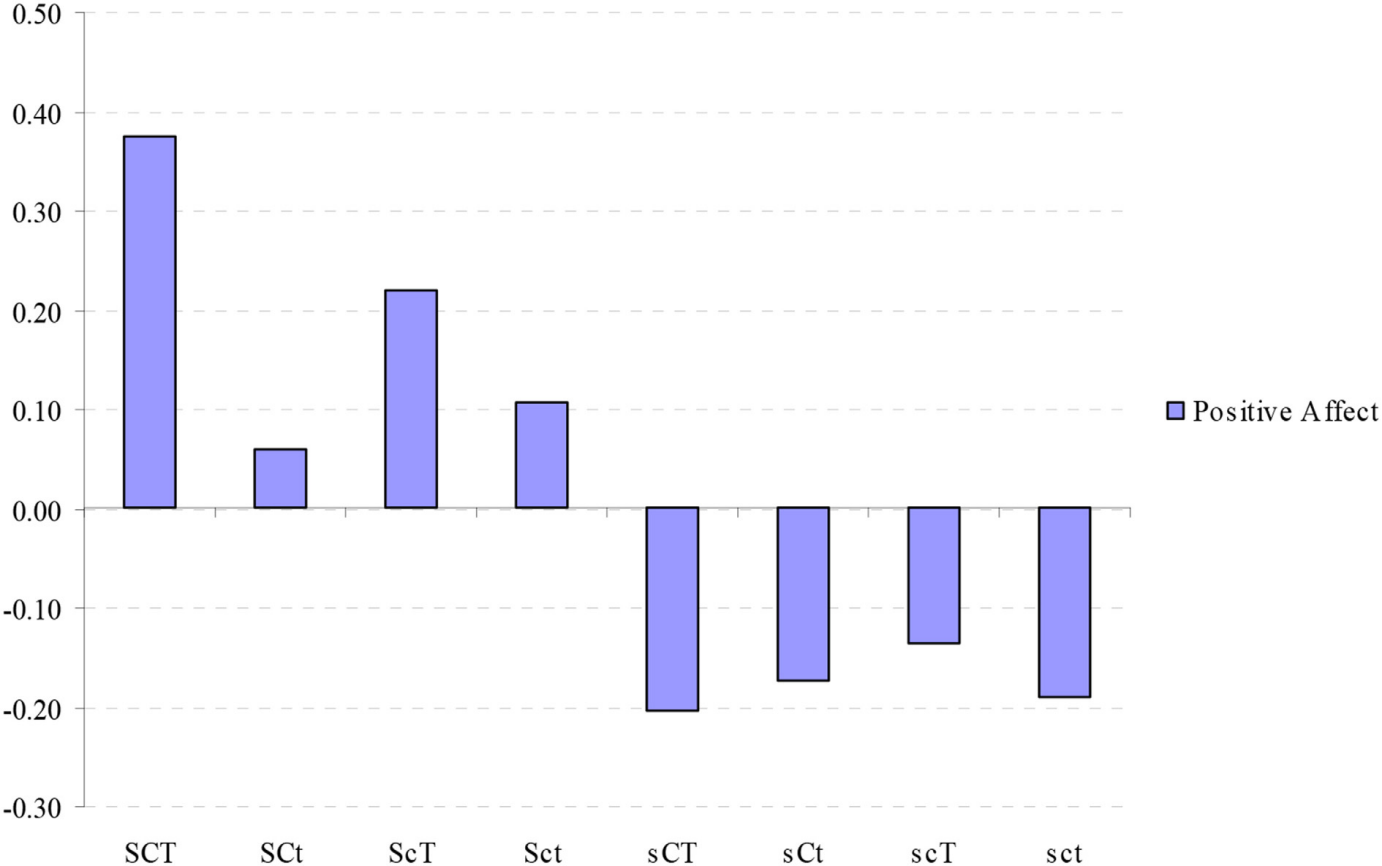
**Standardized values (mean = 0, *SD* = 1) of positive affect in different character combinations**. SCT, creative; SCt, Organized; ScT, Fanatical; Sct, Autocratic; sCT, Moody; sCt, Dependent; scT, Disorganized; sct, Depressive; ANOVA: *F* = 15.89, *p* = 0.00.

The standardized positive affect scores were compared among the participants in the 8 character profiles (Figure 1). Analysis of variance revealed highly significant differences among the groups (*F* = 15.89, *p* = 0.00). Bonferroni corrected comparison between groups showed that the creative (SCT) profile was significantly higher in positive affect than in all other profiles with the exception of autocratic (Sct) and fanatical (ScT) profiles. The depressive profile (sct) was significantly lower in positive affect than creative (SCT) and fanatical (ScT) profiles.

We evaluated the non-linear influence of each of the character dimensions on positive affect by paired comparisons of the effect of extremes of each character dimension when the other two were controlled. Higher Self-directedness was consistently associated with higher positive affect for each of the four possible configurations of Self-Transcendence and Cooperativeness. With regard to Cooperativeness and Self-Transcendence, only the comparison between the creative (SCT) and organized (SCt) profiles reached a statistically significant difference (*t* = 3.83, *p* = 0.00), with the Self-Transcendence associated with higher positive affect (**Table 7**).

### Character profile and negative affect

**Figure 2:**
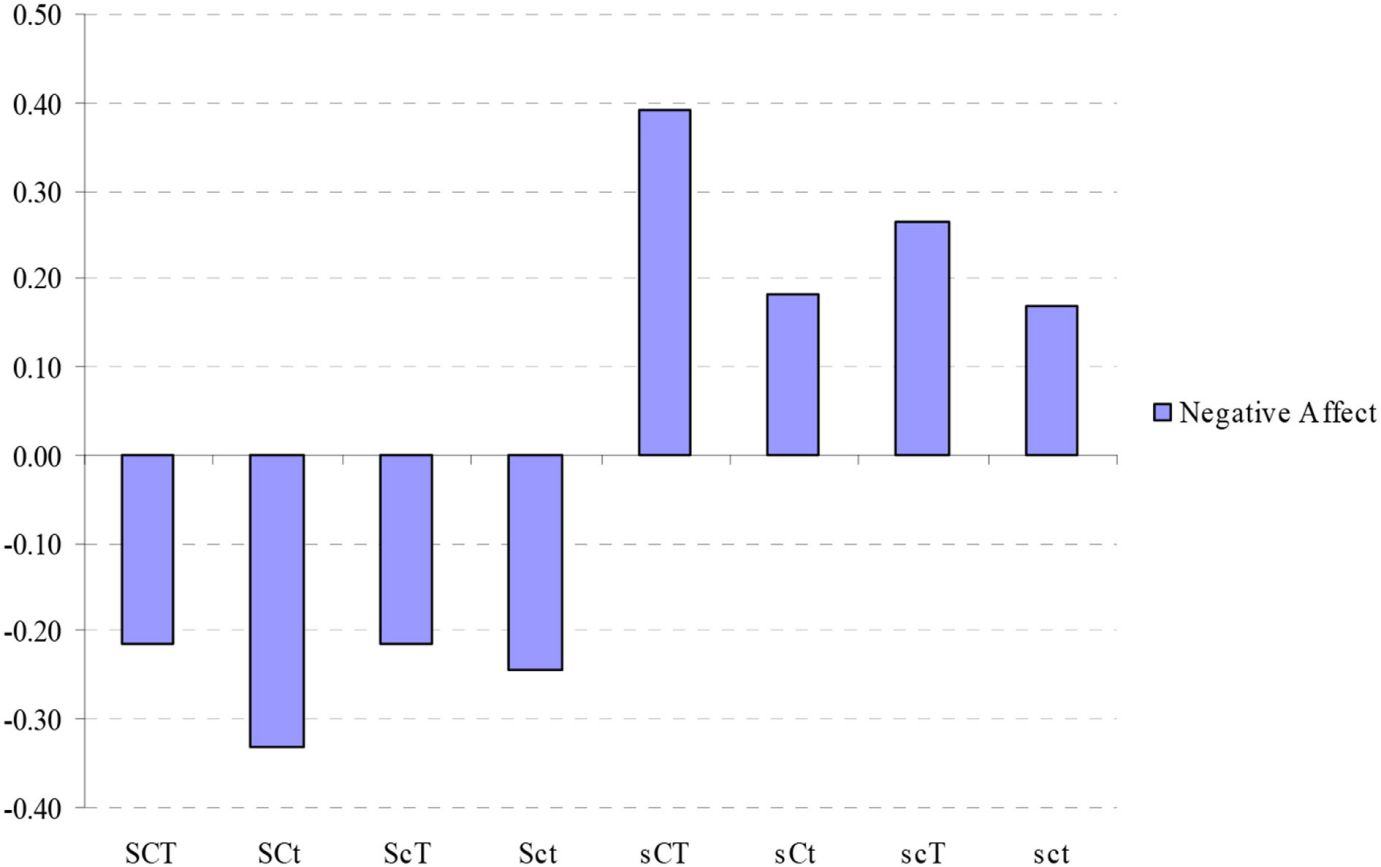
**Standardized values (mean = 0, *SD* = 1) of negative affect in different character combinations**. SCT, creative; SCt, Organized; ScT, Fanatical; Sct, Autocratic; sCT, Moody; sCt, Dependent; scT, Disorganized; sct, Depressive; ANOVA: *F* = 9.84, *p* = 0.00.

Analysis of negative affect variance among the participants in the 8 character profiles showed that the groups were significantly different one from another (*F* = 9.84, *p* = 0.00). Figure 2 shows the standardized scores. Bonferroni range correction showed that the first four character profiles with high Self-directedness [Creative (SCT); Organized (SCt); Fanatical (ScT); and Autocratic (Sct)] were significantly lower than the other four character profiles [Moody (sCT); Dependent (sCt); Disorganized (scT); and Depressive (sct)] with exception of the comparison between fanatical (ScT) and dependent (sCt) profiles (*md* = −0.40, *p* = 0.15).

The evaluation of the non-linear interactions of character dimensions on negative affect showed that the Self-directedness had a significant inverse association with negative affect for each of the four possible configurations of the other two character traits (**Table 7**). Cooperativeness and Self-Transcendence were not associated with lower negative affect in any contrast.

### Character profile and non-affective measures

**Figure 3:**
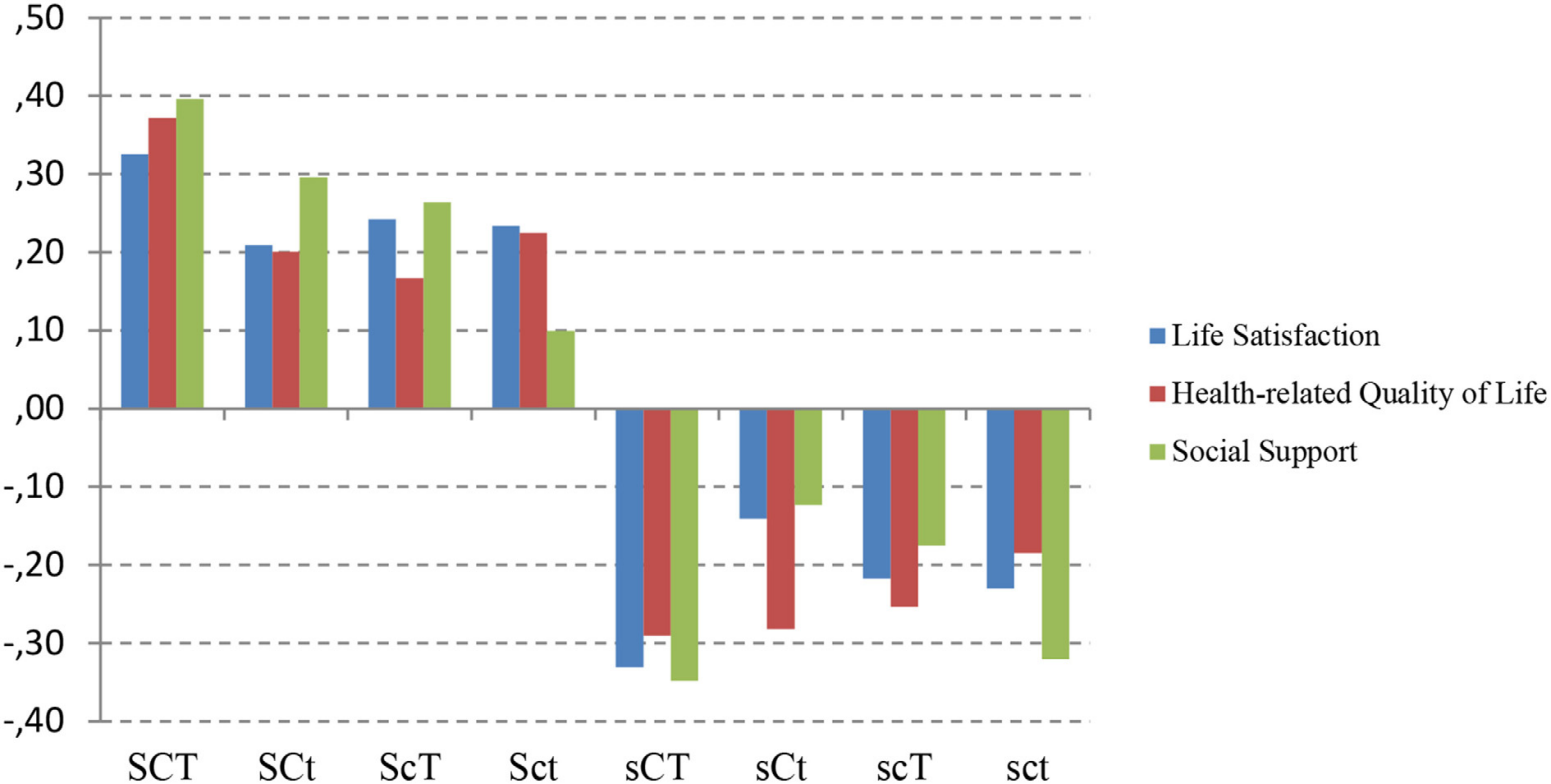
**Standardized values (mean = 0, *SD* = 1) of life satisfaction, health-related quality of life and social support in different character combinations**. SCT, creative; SCt, Organized; ScT, Fanatical; Sct, Autocratic; sCT, Moody; sCt, Dependent; scT, Disorganized; sct, Depressive; All three individual ANOVAs are significant at *p* < 0.05.

The relationships among our non-affective measures of well-being and character profiles were examined (Figure 3 and Table 5). Analysis of variance revealed that the profile groups differed significantly for the three non-affective measures of well-being: life satisfaction (*F* = 14.64, *p* = 0.00), perceived social support (*F* = 19.99, *p* = 0.00) and health-related quality of life (*F* = 15.32, *p* = 0.00). *Post-hoc* group comparisons using the Bonferroni correction showed that for life satisfaction the means of the creative (SCT) and organized (SCt) profiles were significantly higher than those of all profiles that were not high in Self-Directedness. Also the mean of depressed (sct), disorganized (scT) and moody (sCT) profiles were significantly lower than those of all profiles that were high in Self-Directedness. For health-related quality of life, all profiles with high Self- Directedness differed significantly from those with low Self-Directedness, with exception of the contrast between fanatical (ScT) and depressive (sct) profiles. For Life Satisfaction all profiles with high Self- Directedness differed significantly from those with low Self-Directedness, with exception of the contrast between fanatical (ScT) and dependent (sCt) profiles. For Social Support, all profiles with high Self- Directedness differed significantly from those with low Self-Directedness, with exception of the contrast between autocratic (Sct) and disorganized (scT), autocratic (Sct) and dependent (sCt), and between fanatical (ScT) and dependent (sCt) profiles. Profiles with low self-directedness did not differ from each other and neither did profiles with high self-directedness.

**Table 5:** **Comparisons between character profiles in standardized measures of well-being, social support and health-related quality of life**.

	**Life satisfaction**		**Social support**		**Health-related quality of life**	
	***t***	***p***	***t***	***p***	***t***	***p***
**SELF-DIRECTEDNESS**						
SCT vs. sCT	6.87	0.00	7.07	0.00	6.32	0.00
SCt vs. sCt	3.79	0.00	3.84	0.00	4.59	0.00
ScT vs. scT	2.85	0.01	3.59	0.00	3.31	0.00
Sct vs. sct	5.01	0.00	4.71	0.00	4.37	0.00
**COOPERATIVENESS**						
SCT vs. ScT	0.61	0.55	0.99	0.33	1.61	0.11
SCt vs. ScT	−0.32	0.75	0.26	0.80	−0.27	0.79
sCT vs. scT	−1.02	0.31	−1.71	0.09	−0.34	0.73
sCt vs. sct	0.81	0.42	1.90	0.06	−0.88	0.38
**SELF-TRANSCENDENCE**						
SCT vs. SCt	1.67	0.10	1.14	0.25	5.36	0.04
ScT vs. Sct	0.044	0.97	1.27	0.21	−0.43	0.67
sCT vs. sCt	−1.49	0.14	−1.78	0.08	−0.07	0.95
scT vs. sct	0.19	0.90	1.80	0.07	−0.80	0.43

*SCT, creative; SCt, Organized; ScT, Fanatical; Sct, Autocratic; sCT, Moody; sCt, Dependent; scT, Disorganized; sct, Depressive*.

Taking interactions among the character traits into account, higher Self-directedness was associated with greater life satisfaction, health-related quality of life and perceived social support in all contrasts. Cooperativeness and Self-Transcendence had little or no association with any measure of non-affective well-being (Table 5).

### Character profile and composite health index and happiness index

**Figure 4:**
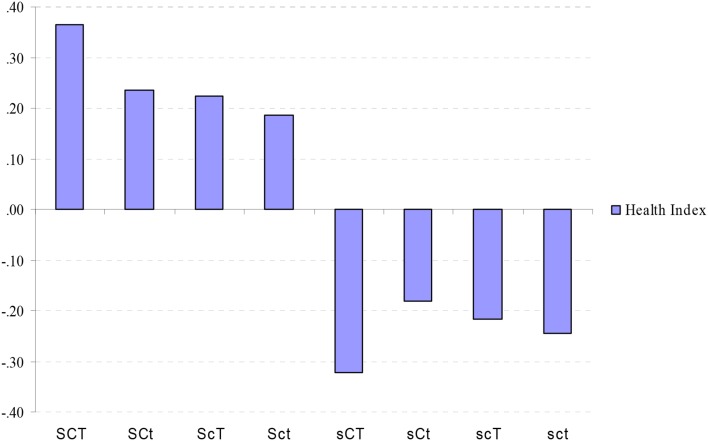
**Standardized values (mean = 0, *SD* = 1) of Composite Health Index in different character combinations**. SCT, creative; SCt, Organized; ScT, Fanatical; Sct, Autocratic; sCT, Moody; sCt, Dependent; scT, Disorganized; sct, Depressive; ANOVA: *F* = 25.53, *p* = 0.00.

The descriptive statistics for Composite Health Indicator (CHI), Hapinness Indicator (HI) and positive and negative affect by character profile are showed in Table 6 and the analysis of variance showed that the profile groups differed on the Composite Health Indicator (CHI) (*F* = 25.52, *p* = 0.00) (Figure 4) and on the Happiness Index (HI) (*F* = 18.76, *p* = 0.00).

**Table 6:** **Descriptive statistics for standardized Composite Health Index (CHI), Happiness Index (HI) and positive and negative affects by character profile**.

	**CHI**		**HI**		**Positive affect**		**Negative affect**	
	**Mean**	***SE***	**Mean**	***SE***	**Mean**	***SE***	**Mean**	***SE***
SCT	0.36	0.05	0.35	0.06	0.38	0.06	−0.22	0.06
SCt	0.24	0.04	0.27	0.05	0.06	0.05	−0.33	0.05
ScT	0.22	0.10	0.27	0.11	0.22	0.10	−0.21	0.12
Sct	0.19	0.05	0.23	0.07	0.11	0.07	−0.24	0.08
sCT	−0.32	0.07	−0.39	0.09	−0.20	0.08	0.39	0.09
sCt	−0.18	0.08	−0.22	0.10	−0.17	0.10	0.18	0.09
scT	−0.22	0.05	−0.26	0.07	−0.14	0.07	0.26	0.08
sct	−0.25	0.04	−0.22	0.06	−0.19	0.06	0.17	0.06

*SCT, creative; SCt, Organized; ScT, Fanatical; Sct, Autocratic; sCT, Moody; sCt, Dependent; scT, Disorganized; sct, Depressive*.

*Post-hoc* group comparisons using Bonferroni range correction showed that profiles with high Self-Directedness are significantly different from those with low Self-Directedness (Table 7). For both non-affective and affective well-being, higher Self-Directedness was strongly associated with higher well-being regardless of the other two character traits. In our study, Cooperativeness and Self-Transcendence were not associated with non-affective or affective well-being, with exception of Creative (SCT) and Organized (SCt) profiles in terms of Composite Health Indicator (HI) and positive affect.

**Table 7:** **Comparisons between character profiles in standardized measures of Happiness Index (HI), Composite Health Index (CHI), and negative and positive affect**.

	**HI**		**CHI**		**Negative affect**		**Positive affect**	
	***t***	***p***	***T***	***p***	***t***	***p***	***t***	***P***
**SELF-DIRECTEDNESS**								
SCT vs. sCT	7.26	0.00	8.22	0.00	−6.07	0.00	5.65	0.00
SCt vs. sCt	4.99	0.00	5.10	0.00	−5.49	0.00	2.28	0.02
ScT vs. scT	4.04	0.00	4.18	0.00	−3.35	0.00	2.72	0.01
Sct vs. sct	4.64	0.00	5.88	0.00	−4.25	0.00	2.95	0.00
**COOPERATIVENESS**								
SCT vs. ScT	0.70	0.49	1.34	0.18	−0.01	0.99	1.26	0.21
SCt vs. ScT	0.43	0.66	0.117	0.91	−1.02	0.31	−0.51	0.61
sCT vs. scT	−1.15	0.25	−1.27	0.21	1.10	0.27	−0.62	0.54
sCt vs. sct	−0.01	0.99	0.73	0.47	0.12	0.90	0.14	0.89
**SELF-TRANSCENDENCE**								
SCT vs. SCt	1.13	0.26	1.99	0.05	1.56	0.12	3.83	0.00
ScT vs. Sct	0.32	0.75	0.37	0.71	0.22	0.83	0.90	0.37
sCT vs. sCt	−1.27	0.21	−1.35	0.18	1.61	0.11	−0.24	0.81
scT vs. sct	−0.42	0.68	0.44	0.67	1.01	0.31	0.57	0.57

*SCT, creative; SCt, Organized; ScT, Fanatical; Sct, Autocratic; sCT, Moody; sCt, Dependent; scT, Disorganized; sct, Depressive*.

### The influence of character profiles on extremes of non-affective well-being

**Figure 5:**
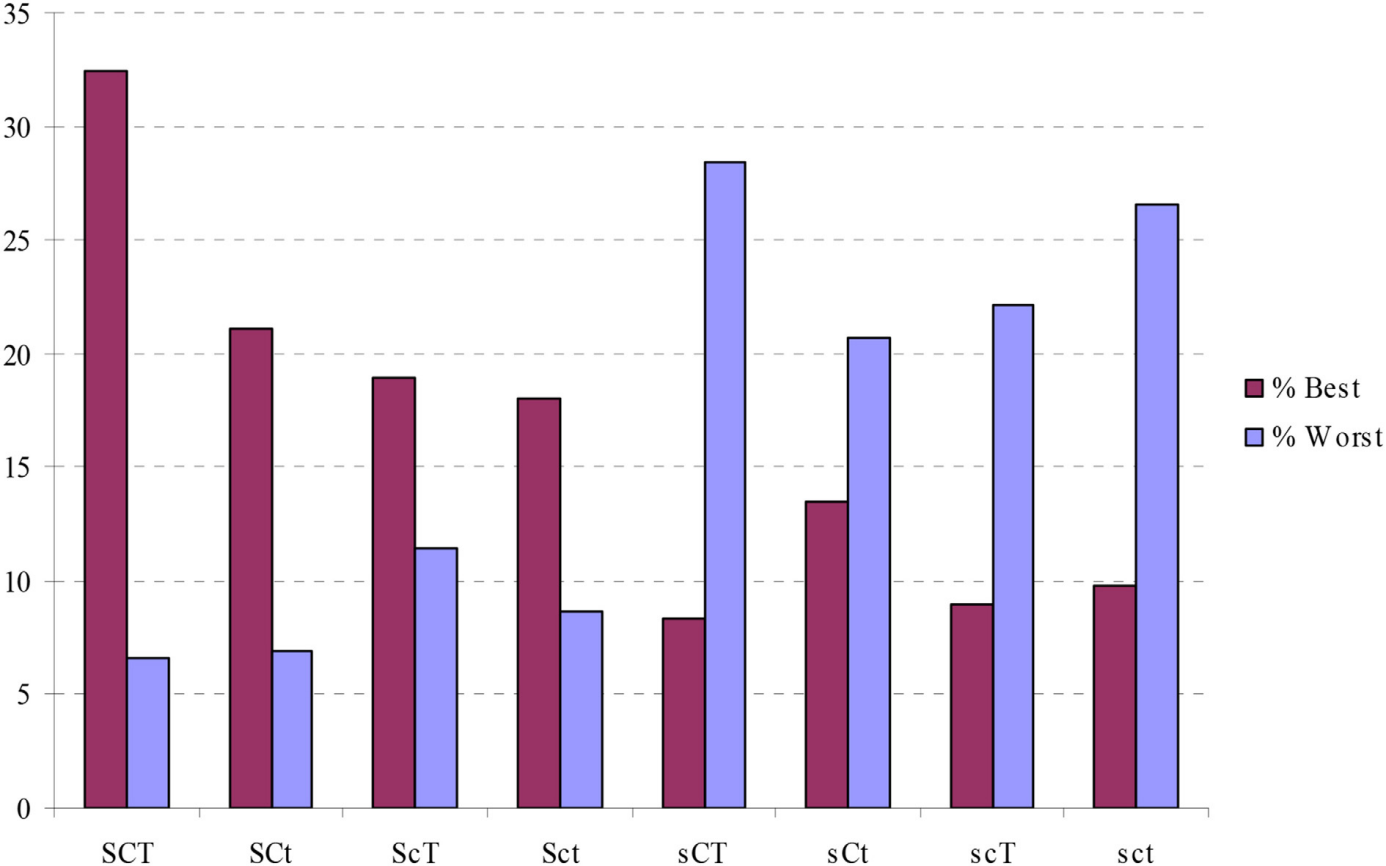
**Percentage of people in each character profile who have “best health” or “worst ill-health.”** SCT, creative; SCt, Organized; ScT, Fanatical; Sct, Autocratic; sCT, Moody; sCt, Dependent; scT, Disorganized; sct, Depressive.

The profile groups differed significantly in the proportion that had extremely “good health” (χ^2^ = 103.61, *df* = 7, *p* = 0.00) and extremely “poor health” (χ^2^ = 62.97, *df* = 7, *p* = 0.00). The percentages with best health and worst health are showed in Figure 5.

In order to quantify the overall linear influence of the three character variables on happiness and wellness, regression analyses were carried out with the Happiness Index (HI) or Composite Health Index (CHI) as the dependent variable predicted by the three character traits. Self-Directedness and Cooperativeness character traits explained a significantly variance of the non-affective well-being, whereas a significantly variance of the affective well-being was explained by Self-Directedness and Self-Transcendence. Self-Directedness (β = 0.43, *t* = 15.24, *p* = 0.00) and Cooperativeness (β = −0.07, *t* = −2.42, *p* = 0.02) explained 15.5% of the variance in Composite Health Index (CHI) (*R*^2^ = 0.16, *F* = 140.70, *p* = 0.00). Self-Directedness (β = 0.32, *t* = 13.24, *p* = 0.00) and Self-Transcendence (β = −0.07, *t* = −2.86, *p* = 0.00) explained 11% in Happiness Index (HI) (*R*^2^ = 0.11, *F* = 89.75, *p* = 0.00).

## Discussion

Multidimensional character profiles are strong predictors of different components of health and of composite wellbeing in adults. In order to describe the influence of character profiles in different components of wellbeing, we estimated the specific contribution of the character traits of self-directedness, cooperativeness and self-transcendence on different aspects of wellbeing (satisfaction with social support, quality of life, life satisfaction and affect). Our results revealed that character profiles have a significant influence on different dimensions of wellbeing, confirming the tendencies found in previous studies with adults (Cloninger and Zohar, 2011; Josefsson et al., 2011).

### Variance on wellbeing measures

Our results confirm that well-being consists of several components of correlated factors. Perceptions of health-related quality of life, social support, life satisfaction and affect are correlated dimensions which need to be taken as whole, in order to achieve a complete understanding of the biopsychological functioning (Cloninger, 2004). Therefore, we estimated two composite indicators: the Composite Health Indicator (CHI, which is the mean of non-affective measures of wellbeing: satisfaction of social support, health-related quality of life and satisfaction with life) and the Happiness Indicator (HI, the score of positive affect minus the score of negative affect). As expected, the composite indicators had a higher variance than individual scales in our study, similarly to what happened with the Israeli and the Finn adults studies (Cloninger and Zohar, 2011; Josefsson et al., 2011). For example, both in the Israeli (CHI = 48.19; Life satisfaction = 41.08; Health quality of life = 24.8 and Social Support = 14.61) and in the Finn (CHI = 74.33; Life satisfaction = 41.73; Health related quality of life = 25.36; Social Support = 60.24) studies, where the CHI variance was superior of the variance of each one of the individual components. In our sample of adolescents, however, the variance of all the wellbeing indicators (both composite and individual indicators) was smaller than in the studies with adults. This was an expected result, as the values of the composite and the values of the individual variables may differ in how they spread out around the mean and around each other. This may suggest that between adolescents the variance on these indicators in smaller than in adults, which may help to understand the specificities found in our sample of adolescents concerning Cooperativeness and Self-Transcendence, and supports the idea that amongst adolescents these processes are not still as mature and differentiated than as they in adults, reason why the variance was smaller in Portuguese adolescents. Additionally, because the range of ages in the adolescents sample (from 12 to 18, mostly and some from 18 to 21) is smaller than in the population representative samples of Israel (24 from 39) and Finland (up 40 years old), and it refers to life stages with substantive qualitative differences, the participants from the adults populations samples were necessarily exposed to different and more heterogenic experiences and contextual influences than adolescents, which may contribute to the less variance on the processes that are expected to co-variate as a function of contextual and experiences heterogeneity.

### Well-being by age and curriculum type

Mean differences between Middle school and High school adolescents revealed statistically significant differences on all dimensions of personality (with exception of Harm Avoidance), and in all indicators of wellbeing (with exception of the Happiness Index). Generally, younger students had higher scores on TCI dimensions of Novelty Seeking and of Self-Transcendence, and higher scores on all the indicators of wellbeing. Exceptions to this tendency were the Happiness Index (no differences) and the Satisfaction with social support (higher scores on older adolescents). Although these results are in line with previous findings on the developmental trends of both TCI dimensions and on Wellbeing indicators, the effect sizes were small for all the significant differences. Concerning curriculum type, statistically significant differences were also found for all the TCI dimensions (with exception of Self-Transcendence) with adolescents enrolled in regular schools presenting lower levels of Novelty Seeking and of Harm Avoidance, but higher values of Reward Dependence, Persistence, Self-Directedness and Cooperativeness. Also, students enrolled in Regular students registered higher levels of wellbeing than their colleagues from Vocational school. For example, adolescents from vocational school registered lower levels of Positive affect and higher levels of negative affect. These results are in line with the expected, as in Portugal typically students in vocational schools registered a relatively poor academic trajectory in regular schools, reason why most of them moved from regular to vocational schools. Again, these statistically significant differences were small, as suggested by the Effect size, and therefore, they need to be considered with caution.

### Influences of personality on wellbeing

As found in studies with adults, in Portuguese adolescents each character trait had a unique contribution to the different dimensions of wellbeing. Self-directedness was significant predictor of life satisfaction, health-related quality of life, perceived social support and positive and negative affect. Cooperativeness and Self-transcendence did not predict positive neither negative affect, but Self-Transcendence was associated with higher positive affect, when associated with high values of Self-Directedness and Cooperativeness. Cooperativeness and Self-Transcendence had little or no linear association with any measure of non-affective well-being.

In Portuguese adolescents, Self-directedness alone explained 15.2% of the variance of non-affective well-being and 9.9% of the variance of the affective well-being. When we used only Cooperativeness to predict well-being, it explained 2.7% of the variance in non-affective well-being and 1.6% of the affective well-being. Self-transcendence had a negligible impact on well-being in linear regression analysis. These results are in line with those found in previous studies with adults. In the Israeli study, Self-directedness alone explained 32% in non-affective well-being and 45% in affective well-being (Cloninger and Zohar, 2011). Cooperativeness explained 4% of the variance of non-affective well-being. In the Finn study, Self-directedness explained 30% of the variance of non-affective well-being and 40% of the variance of affective well-being; Cooperativeness explained 14% in non-affective well-being and 24% in affective well-being (Josefsson et al., 2011). Self-directedness explained higher percentage of non-affective and affective well-being in adults than in Portuguese adolescents. Cooperativeness explained similar percentages of non-affective well-being in Portuguese adolescents (2.7%) and on Israeli adults (4%), but explained a significantly higher percentage in Finn adults (14%), suggesting that Cooperativeness in less important in predicting well-being in Portuguese adolescents and in Israeli adults. Besides, it suggests that the impact of Cooperativeness in predicting well-being depends on both cultural and developmental factors. Self-transcendence had a negligible effect on well-being in linear regression analysis in the previous studies with adults (Cloninger and Zohar, 2011; Josefsson et al., 2011) and with adolescents (Garcia, 2011; Garcia and Moradi, 2011). Character dimensions of Self-directedness and Cooperativeness explained 15.5% of the variance in non-affective well-being in Portuguese adolescents and Self-Directedness and Self-Transcendence explained 10.6% in affective well-being. In the Israeli study, character dimensions explained 36% of the variance in non-affective well-being and 45% in affective well-being (Cloninger and Zohar, 2011). In the Finn study TCI character dimensions taken together explained 56% of the variance of non-affective well-being and 65% in affective well-being (Josefsson et al., 2011).

*Self-directedness* was strongly associated with all aspects of well-being, even when interacting with the other character dimensions. In fact, the shift between the valence of the different indicators taken separately (positive affect, negative affect, satisfaction with life, health-related quality of life, satisfaction with social support, and from predominant good health to ill health), happened between autocratic (Sct) and moody (sCT) profiles. Self-directedness refers to the person understanding of himself or herself as an autonomous individual, with responsibilities, purposes and resources. The individuals' awareness of his or her responsibilities, purposes and resources regulates people's hopes and desires. Individuals with high Self-directedness are responsible, purposeful, and resourceful and with habits congruent with long term goals (Cloninger et al., 1993), which influences strongly physical, mental and social well-being (Cloninger and Zohar, 2011; Josefsson et al., 2011). Because of the typical challenges of individualistic and performance oriented societies, adolescents are especially encouraged to develop self-directedness processes. Several outcomes in adolescence (including academic achievement) are strongly predicted by aspects of Self-directedness (such as self-discipline) (Duckworth and Seligman, 2005), and of Persistence (Moreira et al., 2012b), more than by Intelligence Quotient (IQ). Consequently, society (including family and school) tend to emphasize more the development of the processes involved in self-directedness, because of its important to objective outcomes in present and future life (such as academic achievement, occupational outcomes,) rather than aspects of Cooperativeness or Self-transcendence. Additionally, studies conducted by Garcia and colleagues showed that self-directedness mediates the influence of persistence on adolescents' positive affect (Garcia et al., 2012), emphasizing the importance of self-directedness in modulating the expression of dispositional tendencies in adaptive functioning. Self-directed adolescents tend to have good habits and regulate their behaviors accordingly to their long-term goals, which tend to result in long-term achievements, in positive rewards and in positive evaluations of several aspects of individuals' lives, and therefore to well-being.

*Cooperativeness* was found to explain a variance of well-being similar to what happened in Israeli adults, but less than in Finn adults. Also in adults, Cooperativeness was associated with the perception of social support, increased non-affective well-being and to reduced negative emotions (Cloninger and Zohar, 2011; Josefsson et al., 2011). However, in Portuguese adolescents, those results were not found. Cooperativeness refers to the individuals' awareness of being part of a society. Individuals high cooperative are empathic, helpful and social tolerant (Cloninger et al., 1993). Because of this, the importance of cooperativeness in the satisfaction of social support is associated to the role cooperativeness plays in being well succeeded in having social support. In childhood and adolescence, social support is highly dependent of the surrounding and established social networks (such as family, school, etc.). Being social support associated to the satisfaction of the individuals' needs, western societies are organized so adults guarantee the satisfaction of child and adolescents basic needs in a collective responsibility perspective. Societies are organized in a way by which several factors are present, regardless of the child and adolescents characteristics (a child or an adolescent should have the need support at home, school, etc., regardless of being more or less cooperative). As the individual grows, he or she becomes more autonomous, which tend to mean that the individual is more dependent of his or her characteristics to be well succeed in adaptation. Therefore, more cooperative individuals are more likely to be more effective in creating, feeding and mobilizing social networks, which increases the probability of having his or her needs satisfied and of perceiving the social support as satisfactory.

*Self-transcendence* was found to have a negligible linear association with well-being in Portuguese adolescents, but when in interaction with Self-directedness it predicted 10.4% of the variance of affective well-being. Although less than in Israeli adults, the non-linear influence of Self-transcendence in well-being was similar to what was found in Finn adults. Self-transcendence refers to the awareness of being part of a whole, where all things, people and animals are connected (Cloninger et al., 1993). Abstractedness significantly increases during adolescence, allowing individuals for developing a growing awareness of self-transcendent aspects. Although self-transcendence aspects are involved in adolescents' mapping of the existence, they are typically more centered in their concrete aspects of experience (self-image, peer relations, etc.). As a consequence, concrete aspects of existence are more salient in adolescents' experiences than more abstract and transcendent aspects. Therefore, it is understandable that adolescents' evaluations of the several aspects of life be more dependent of more concrete and immediate factors, rather than of transcendent aspects, meaning that self-transcendence play a more distal influence on adolescents' wellbeing, with self-directedness aspects playing a more proximal influence on adolescents' wellbeing. Besides, self-transcendence and abstractedness become more differentiated in late adolescence and early adulthood. The mean age of the participants on our studies was about 15 years old, an age where it is expected that a significant maturation and differentiation of self-transcendence is still to occur. Therefore, self-transcendent aspects may have a more distal impact on adolescents' wellbeing, when compared with self-directedness. Previous studies with adults revealed that, when the interactions among character traits are taken into account, Self-transcendence had a consistent impact on the presence of both positive and negative emotions (Cloninger and Zohar, 2011; Josefsson et al., 2011). Also in adolescents, when the other two character traits were held constant, Self-transcendence increased non-affective well-being and both positive and negative affect. In spite of the cultural differences found in the studies of Israel and Finland, and of the differences between our study with adolescents and the two adult studies, the three studies assessing the non-linear associations between well-being and multidimensional character profiles registered similar tendencies.

*Altogether*, these results suggest that Self-directedness is a significant predictor of well-being, regardless of the culture and age, and that cooperativeness and self-transcendence influences on well-being depends on cultural, religious and developmental factors. In fact, In the Finn data all associations of Cooperativeness with happiness, composite health or affect were significant (Josefsson et al., 2011), but no such associations were observed in the Israeli study (Cloninger and Zohar, 2011), suggesting that Cooperativeness may be a more important predictor of affective and non-affective well-being in Finland than in Israel (Josefsson et al., 2011). Similarly, our results suggest that Cooperativeness and Self-transcendence may be less important in predicting affective and non-affective well-being in adolescents than in adults. In spite of this, and because cultural differences were found in Finland and Israel, cross-cultural studies exploring the associations between the multidimensional profiles of Character are needed in order to confirm the trends found in Portuguese adolescents. Configurations of personality dimensions allow for the multidimensional nature of adaptive human functioning (Cloninger and Zohar, 2011), and are more compatible to the interdependence of the different components of heath. Similarly, well-being is a multicomponent phenomenon, also because it depends on the dynamics between the different individuals functioning domains involved in adaptation. As found in previous studies, this study reveals that well-being depends on specific interactive and non-linear dynamics of personality development (Cloninger and Zohar, 2011), and that each dimension of well-being must be considered as an interdependent domain involved in the individuals' adaptive functioning (Cloninger, 2009).

This was the first study replicating in adolescents the two national-based studies which assessed the non-linear interactions between character dimensions in the explanation of well-being in Israeli and Finn adults (Cloninger and Zohar, 2011; Josefsson et al., 2011). Our results clearly confirm that configurations of the three dimensions of character measured by the Temperament and Character Inventories (both the adolescent and adult versions) influence affective and non-affective well-being also in adolescents. The person-centered approach used in this study is more consistent with the holistic and dynamic nature of human beings, allowing for an understanding of human development within an individual. Also, results from this study confirm the importance a non-linear approach to the relation between personality and well-being, as the non-linear impact of Cooperativeness and Self-transcendence on different aspects of wellbeing would not be captured with linear regression analysis only.

#### Implications

*Adolescence* is a developmental period characterized by marked changes in adolescents in cognition, emotion, behavioral and contexts. These changes result from the process of maturation and differentiation of neuropsychological systems, including the behavioral activation, the inhibition, the reward dependence systems and the higher order cognitive self-regulatory processes. These specific neuroanatomical and functional systems change over time, with emotional and cognitive dimensions presenting distinct patterns of development over the lifespan (Josefsson et al., 2013a). Adolescence is characterized by higher sensitivity to novelty, exploration and to reward, and lower inhibitory control (Eldreth et al., 2013). Adolescents' behaviors result from the interaction between the changing/maturating neuroanatomic circuitries and processes and contextual influences (Josefsson et al., 2013b). The unbalance between the different systems, and depending on the dynamics between neuropsychological systems and context characteristics (specifically the failure of higher order regulatory processes in modulating the adaptive expression of the emotional responses and behaviors) place adolescents at increased risk for poor functioning and for maladaptive developmental trajectories, including risk behaviors and emotional lability. These patterns of functioning are significant components of developmental cascades, which are strong predictors of functioning also during adulthood (Eldreth et al., 2013). Conversely, an adaptive maturation of higher order cognitive processes is a strong predictor of healthy personality development and of healthy functioning, including less psychopathology and more agentic motivation (Moreira et al., 2014a). Additionally, different higher order cognitive processes (self-directedness, cooperativeness and self-transcendence) are involved in the individuals' psychobiological organizations underlying behavior. Our results confirm that also in adolescents different combinations of character dimensions are strong predictors of both negative and positive functioning, with elevation in the three dimensions being associated with healthy functioning, similar to what was found with adults (Cloninger and Zohar, 2011; Josefsson et al., 2011).

Because adaptive functioning results from the dynamics of developmental cascades, and because positive aspects are crucial for healthy developmental trajectories, the understanding of the developmental associations between personality and well-being is highly relevant for the promotion of youth positive developmental trajectories promotion.

Almost half of the European adolescents report multiple health complaints, poor to fair health, low life satisfaction or a combination of these (Ravens-Sieberer et al., 2009). Because of the associations of well-being with adaptive and maladaptive functioning, these results suggest that besides the promotion of educational persistance and motivational dimensions (Walker et al., 2006; Moreira et al., 2013), the promotion of well-being needs to be established as an educational priority, as it is an avenue for the promotion of a healthy psychobiological adaptation to experience. On the one hand, although life circumstances also influence long-term levels of well-being, personality explains a significant portion of the variance of well-being (Diener et al., 2003; Suldo and Shaffer, 2008). Several aspects of positive mental health operationalized by Vaillant (2012) refers to and are well predicted by Cloninger' character dimensions of Self-directedness, Cooperativeness and Self-Transcendence. Therefore, schools need to accept their responsibility in promoting adaptive trajectories promotion (rather than focus on the moment, on deficits or in grades), which requires the promotion of healthy personality development. In fact, as highlighted by Heldon and Lybormirsky, sustainable happiness is possible through intentional activity changes, more so than through circumstantial changes (Heldon and Lybomirsky, 2006; Blustein, 2008; Hosie and Sevastos, 2010). This fact justifies systematized, internationalized and continued school-based approaches to the promotion of mental health, including well-being (Cloninger et al., 2010; Moreira et al., 2014b). On the other hand, there is a robust body of evidences about the efficacy of school-based strategies for the promotion of higher cognitive self-regulatory functions. These strategies are been called by several names, including social and emotional skills, emotional intelligence or socio-emotional learning (Moreira et al., 2012a). Programmes for the promotion of these dimensions are efficient in promoting social and emotional skills, positive attitudes and behaviors (Kimber et al., 2008; Moreira et al., 2010), including positve academic trajectories (Durlak et al., 2011).

Schools are a privileged avenue for the promotion of youth positive development. In order to be effective in promoting youth positive development, schools need to incorporate in their objectives and practice the promotion of a healthy personality development.

#### Limitations

This study has some limitations. The instruments used in this Portuguese adolescents study, although age-appropriate, are not exactly the same used in both previous studies in Israel and Finland. Another limitation is that this study used a cross-sectional sample, which prevents us of establishing causal relations. However, the associations between multidimensional profiles of character and well-being were replicated in three samples (two adult samples and one adolescent sample), which suggest that these trends are consistent. Future studies that replicate this study in other cultures are needed, in order to test the associations between configurations of character dimensions and well-being in adolescents from different cultures.

### Conflict of interest statement

The authors declare that the research was conducted in the absence of any commercial or financial relationships that could be construed as a potential conflict of interest.
